# Role of working conditions in the explanation of occupational inequalities in work injury: findings from the national French SUMER survey

**DOI:** 10.1186/s12889-018-5254-7

**Published:** 2018-03-12

**Authors:** Isabelle Niedhammer, Thomas Lesuffleur, Géraldine Labarthe, Jean-François Chastang

**Affiliations:** 1 INSERM, U1085, Research Institute for Environmental and Occupational Health (IRSET), Epidemiology in Occupational Health and Ergonomics (ESTER) Team, Faculté de Médecine, 28 rue Roger Amsler, CS 74521, F-49045, Angers, Cedex 01 France; 2 University of Angers, Epidemiology in Occupational Health and Ergonomics (ESTER) Team, Angers, France; 3 Ministry of Labour, DARES, Paris, France

**Keywords:** Work injury, Social inequalities in health, Working conditions, Occupational exposures, Psychosocial work factors

## Abstract

**Background:**

Social inequalities in work injury have been observed but explanations are still missing. The objectives of this study were to evaluate the contribution of working conditions in the explanation of social inequalities in work injury in a national representative sample of employees.

**Methods:**

The study was based on the cross-sectional sample of the national French survey SUMER 2010 including 46,962 employees, 26,883 men and 20,079 women. The number of work injuries within the last 12 months was studied as the outcome. Occupation was used as a marker of social position. Psychosocial work factors included various variables related to the classical job strain model, psychological demands, decision latitude, social support, and other understudied variables related to reward, job insecurity, job promotion, esteem, working time and hours and workplace violence. Occupational exposures of chemical, biological, physical and biomechanical nature were also studied. Weighted age-adjusted Poisson regression analyses were performed.

**Results:**

Occupational gradients were observed in the exposure of most psychosocial work factors and occupational exposures. Strong occupational differences in work injury were found, blue-collar workers being more likely to have work injury. Chemical, biological, physical and biomechanical exposures contributed to explain the occupational differences in work injury substantially. Noise, thermic constraints, manual materials handling, postural/articular constraints and vibrations had significant contributions. Psychosocial work factors also contributed to explain the differences especially among women.

**Conclusion:**

Prevention policies oriented toward chemical, biological, physical, biomechanical and psychosocial work exposures may contribute to reduce the magnitude of occupational differences in work injury.

## Background

Work injury represents a major burden for the society and companies because of their substantial costs and related absenteeism and disability [1, 2]. Studies reported that low-skilled and manual workers were more likely to have work injury [3–6]. These findings are in line with the results provided by social epidemiology studies that underlined social inequalities in various health outcomes [5], including injury in general [6]. Nevertheless, the literature appears sparse on the topic of social inequalities in work injury specifically and still more seldom on the factors that may contribute to explain these inequalities.

Working conditions play an important role in the occurrence of work injury. However, the contribution of working conditions and occupational exposures to social inequalities in work injury has been studied very rarely to date. One exception may be one of our previous studies that explored work injury among other health-related outcomes and was not focused on work injury exclusively [7]. It is thus difficult to evaluate the role of working conditions and occupational exposures in the explanation of social inequalities of work injury.

According to Eurostat, a large part of nonfatal work injuries result from physical and biomechanical exposures at the workplace [2]. Etiological studies identified a number of occupational exposures that increase the risk of work injury, such as for example physical demands [8, 9], noise [10, 11], heat [12], shift/night work and long working hours [13]. Psychosocial work factors may also play a role in work injury. These factors have been defined using theoretical models, the most used being the job strain model [14] composed of three main dimensions: psychological demands, decision latitude, including both skill discretion and decision authority, and social support from colleagues and supervisors. The combination of high psychological demands and low decision latitude (job strain) may have adverse effects on health, and these effects may be increased by low social support (iso-strain). Another model, the effort-reward imbalance model, defines the imbalance between high effort spent at work and low reward received (in terms of esteem, job promotion and job security) [15]. Other psychosocial work factors have emerged more recently in the literature: workplace violence such as physical violence, sexual harassment, verbal abuse and bullying, predictability as well as demands for responsibility. Studies showed that high psychological demands, low decision latitude, low social support and/or job strain were associated with work injury [16–22]. A few studies found significant associations between low reward [19], workplace violence/conflicts [16, 19, 21–23] and work injury.

As poor working conditions and occupational exposures were found to be associated with work injury and as these conditions and exposures may be more prevalent among low-skilled and manual workers [7, 24], they may be considered as pertinent explanations of social inequalities in work injury. Work injury is an avoidable outcome, consequently information on this topic may be crucial to prevent work injury and reduce social inequalities in this outcome.

This study aimed at exploring occupational differences in work injury and at evaluating the contribution of a large number of occupational exposures of psychosocial, chemical, biological, physical and biomechanical nature in the explanation of these differences.

## Methods

### Study population

The SUMER survey is a periodic national cross-sectional survey from two departments of the French ministry of labour conducted every seven years. Its objective is to evaluate occupational exposures among the national working population of employees, in order to define preventive strategies and research priorities in France. The SUMER survey is based on a network of voluntary occupational physicians, in charge of compulsory medical examinations of employees, who collect the data for a random sample of their employees. Each occupational physician selected 30 employees of the population of employees seen during the period of collection using a random method (one employee of 10 or 20 for example). Occupational medicine is mandatory for all employees in France; consequently, every employee has a medical examination with an occupational physician periodically. SUMER 2010, the last survey conducted in 2010, included around 50,000 employees interviewed about their physical, biological, chemical, biomechanical, organizational and psychosocial exposures by 2400 occupational physicians. The survey included two questionnaires: a main questionnaire and a self-administered questionnaire. The occupational physicians filled in the main questionnaire mainly about physical, biological, chemical, biomechanical and organizational exposures for each employee. Employees filled in a self-administered questionnaire in which their responses were collected about psychosocial work factors and health outcomes. Several articles have already been published by our team using these survey data [19, 25–29].

### Work injury

The information about work injury was collected in the self-administered questionnaire. Work injury was measured by the number of injuries (0, 1, 2, 3 or more), which required a medical treatment and at least one day of absence within the last 12 months. We used the number of work injuries within the last 12 months as the outcome of our study.

### Psychosocial work factors

Psychosocial work factors were constructed using the data collected in the self-administered questionnaire.

Job strain model dimensions were constructed using the validated French version of the questionnaire [30, 31]: decision latitude (9 items, Cronbach alpha = 0.78, including 6 items for skill discretion and 3 items for decision authority), psychological demands (9 items, Cronbach alpha = 0.80) and social support (8 items, Cronbach alpha = 0.82, including 4 items for social support from colleagues and 4 items for social support from supervisors). The scores were constructed according to the recommendations by Karasek and dichotomized at the median of the total sample. Job strain was defined by the combination of high demands and low latitude, and isostrain by the combination of job strain and low support.

Reward (11 items, Cronbach alpha = 0.85, including 5 items for esteem, 2 items for job security and 4 items for job promotion) from the effort-reward imbalance model was measured using the validated French version of this questionnaire [32]. Reward and its sub-dimensions were dichotomized at the median of the total sample.

Five working time variables were studied: long working hours (1 item, ≥48 h/week following the European directive on working time), night work (1 item, working between 12 and 5 am ≥1 night/week), shift work (1 item, either permanent or alternating/rotating shifts), unsociable work days (1 item, working on Sunday or Saturday ≥1 day/week), and predictability of schedules (4 items: information about time schedules for the next day, week, month and the next three months).

Three factors were related to workplace violence: physical violence or sexual assault (2 items), bullying (9 items) and verbal abuse (2 items). Exposure was defined by at least one situation of workplace violence for each factor.

Demands for responsibility (4 items: a mistake in work may lead to serious consequences for product/service quality, to serious financial losses for the company, to dangerous consequences for the safety of people or oneself, and to wage/work/job sanctions for oneself) was dichotomized at the median of the total sample.

### Other occupational exposures

Other occupational exposures (physical, biomechanical, biological and chemical exposures) were measured by the occupational physicians using their expert evaluation and collected in the main questionnaire.

Physical exposure was defined by at least 20 h of exposure to noise, thermic constraints, radiations or controlled air/space within the previous week.

Biomechanical exposure was defined by at least 20 h of exposure to manual materials handling, postural/articular constraints, vibrations or driving within the previous week.

Biological exposure was defined by at least one biological exposure within the previous week.

Chemical exposure was defined by at least one chemical exposure within the previous week.

The questionnaires and the evaluation of all occupational exposures were built using national and European guidelines and a full description may be found elsewhere [21].

### Occupation

Occupation was coded using the French national classification of occupations (PCS by INSEE) that is close to the International Standard Classification of Occupation (ISCO), and was used as a measure of social position and included at the first level of the classification four categories of employees: professionals/managers used as the reference category, associate professionals/technicians, clerks/service workers, and blue-collar workers. Occupation was used as a marker of social position as it characterizes adult social position, is available for all workers, and may reflect occupational exposures better than education or other markers [33, 34].

### Statistical methods

The data were weighted to provide estimates which were nationally representative of the French working population of employees (i.e. 22 millions of employees representing 92% of the total national population of employees in France, excluding the public sector of education and some ministries). The method for the calculation of weights performed by the DARES of the French ministry of labour had different objectives: to control for the potential bias related to volunteering of occupational physicians by taking into account their characteristics in comparison with the characteristics of the national population of occupational physicians, to control for the potential bias related to the differential periodicity of medical examinations (highly exposed employees have more frequent medical examinations), to control for the potential bias related to non-response to the survey, and finally to provide final weights using a calibration on margins to take the characteristics of the national French population of employees into account. These final weights were calculated using the following calibration variables: gender, age, nationality, working time (full or part time), occupation, company size, and economic activity. All analyses were performed using weighted data.

Major differences in work injury are usually observed between gender and age groups, the prevalence of work injury is lower among women than among men, and may decrease with age [2]. Men and women were analyzed separately and age was taken into account in all models.

The statistical analysis included three steps:

- Firstly, the study sample was described and the differences between occupations for all variables were tested using Rao-Scott Chi-Square test to take the weights into account.
- Secondly, the associations between occupation and work injury were studied using weighted Poisson regression analysis, work injury being the dependent variable. The contributions of psychosocial work factors and other occupational exposures to occupational inequalities in work injury were calculated for the three occupational groups: associate professionals/technicians, clerks/service workers and blue-collar workers in comparison to professionals/managers. The contribution of each work factor (or a set of work factors) to the explanation of the occupational differences was estimated by the Karlson, Holm and Breen method (KHB) [35, 36], that allows to compare the estimated coefficients of two nested nonlinear probability models. Positive contributions (%) indicated rate ratio (RR) reductions and negative contributions indicated RR increases. A 95% confidence interval was calculated for each contribution using the Jackknife method to provide the significance of each contribution.

Several models were performed:

- A first model included only occupation and age as independent variables (model 0).
- Each psychosocial work factor or occupational exposure was added separately to model 0 (extended model 0).
- All the psychosocial work factors that displayed significant positive contributions for at least one gender or occupational group were added simultaneously to model 0 as independent variables in model 1 and model 2.
- Similarly, all the occupational exposures that displayed significant positive contributions for at least one gender or occupational group were added simultaneously to model 0 as independent variables in model 3 and model 4.
- Finally, all work factors that displayed significant positive contributions for at least one gender or occupational group were added simultaneously to model 0 as independent variables in model 5 and model 6.

Models 1, 3 and 5 included the main dimensions of psychosocial work factors and occupational exposures and models 2, 4 and 6 included their sub-dimensions.

Additional analyses were performed to disentangle the respective contribution of each factor in models 1–6 using the KHB decomposition method that provides unbiased decompositions in the context of nonlinear probability models [37].

- Thirdly, the associations between occupational exposures and work injury were explored. The results for the associations between psychosocial work factors and work injury were presented in a previous paper and showed that high psychological demands, low social support (especially from supervisors), low reward and its sub-dimensions, low predictability, physical violence, bullying and verbal abuse were associated with work injury [19]. The associations between the other occupational exposures and work injury were derived from the models above and presented in the present study.

All statistical analyses were performed using SAS and STATA.

## Results

Of the 53,940 employees asked to participate to the SUMER survey in 2010, 46,962 employees, 26,883 men and 20,079 women, agreed. The response rate was 87%. The description of the sample among men and women may be found elsewhere [19, 25–29]. Almost all psychosocial work factors displayed significant occupational gradients (Table 1), with a higher prevalence of exposure among low-skilled occupational groups (clerks/service workers and/or blue-collar workers): low decision latitude, low social support, job strain, isostrain, low reward (for men only), night work, shift work, unsociable work days, low predictability, the different forms of workplace violence and demands for responsibility (these two last factors especially for men). Two psychosocial work factors displayed inverse occupational gradients; high psychological demands and long working hours were more prevalent among professionals/managers. Marked occupational gradients were observed for the other occupational exposures. Blue-collar workers were more likely to be exposed to physical, biomechanical and chemical exposures and service workers were more likely to be exposed to biological exposures. The Rao-Scott Chi-Square values showed that the magnitude of the occupational differences may be stronger for these exposures than for psychosocial work factors. There was a strong occupational gradient in the prevalence of work injury; blue-collar workers were more likely to have work injury, and the differences between occupations were particularly marked among men.

**Table 1:** Prevalence of psychosocial work factors, other occupational exposures and work injury according to occupational groups

	Professionals, managers	Associate professionals, technicians	Clerks, service workers	Blue-collar workers	Chi-2 value *p*-value
Men (N)	5082	6408	3574	11,819	
Prevalence (%)					
Low decision latitude	20.3	38.0	62.1	56.8	929***
Low skill discretion	21.9	38.1	63.6	55.7	797***
Low decision authority	41.2	54.7	70.0	69.0	517***
High psychological demands	65.6	50.5	37.7	36.7	515***
Low social support	38.6	40.7	43.4	44.2	19***
Low social support (from supervisors)	40.5	40.1	42.7	44.1	13**
Low social support (from colleagues)	64.8	66.2	65.8	66.3	1
Job strain	13.6	19.9	24.8	22.0	83***
Isostrain	9.1	13.2	16.5	14.5	47***
Low reward	44.9	51.4	53.4	48.8	29***
Low esteem	42.8	46.9	50.1	46.0	19***
Job insecurity	44.7	44.0	41.6	44.4	4
Low job promotion	36.7	46.4	48.4	43.0	59***
Long working hours	25.2	9.0	5.1	4.1	798***
Night work	1.2	3.4	8.8	8.0	205***
Shift work	3.5	12.0	23.7	23.0	489***
Unsociable work days	13.5	14.7	32.1	17.8	304***
Low predictability	31.3	31.4	36.4	33.3	14**
Physical violence	0.8	1.4	5.6	1.3	166***
Bullying	19.8	22.6	25.1	21.7	16**
Verbal abuse	16.1	22.9	34.5	14.4	378***
Demands for responsibility	49.2	52.3	45.0	61.4	173***
Biological exposure	6.7	14.2	28.7	15.2	345***
Chemical exposure	6.9	24.9	26.5	60.6	2321***
Physical exposure	21.1	41.6	45.4	78.3	2144***
Noise	14.8	31.4	29.9	65.4	1913***
Thermic constraints	6.1	19.7	30.2	44.5	1040***
Radiations	3.1	5.0	2.1	5.7	48***
Controlled air/space	30.3	19.7	18.5	10.0	428***
Biomechanical exposure	27.1	36.4	44.7	74.0	1727***
Manual materials handling	8.0	29.1	36.7	68.6	2556***
Postural/articular constraints	49.6	63.1	77.0	90.3	1169***
Vibrations	1.3	8.1	8.0	39.6	2020***
Driving	37.6	46.2	35.6	56.7	324***
Work injury					195***
0	99.0	96.2	92.6	89.0	
1	1.0	3.1	6.7	9.7	
2	0.0	0.7	0.6	1.1	
> =3	0.0	0.0	0.1	0.2	
Women (N)	2811	5666	9311	2291	
Prevalence (%)					
Low decision latitude	24.6	46.0	66.9	79.3	1095***
Low skill discretion	27.3	47.1	68.7	78.3	944***
Low decision authority	42.4	62.8	74.1	80.3	497***
High psychological demands	65.7	50.4	41.5	36.6	255***
Low social support	37.6	39.9	40.0	49.4	33***
Low social support (from supervisors)	38.8	41.9	39.3	46.4	19***
Low social support (from colleagues)	61.6	63.7	63.4	70.6	19***
Job strain	16.7	24.7	28.7	30.0	84***
Isostrain	10.6	15.6	17.7	21.7	57***
Low reward	46.6	51.1	49.1	50.4	6
Low esteem	43.2	45.2	42.9	44.8	4
Job insecurity	42.1	42.8	37.0	44.9	33***
Low job promotion	39.1	46.9	47.3	44.2	22***
Long working hours	11.8	2.5	1.6	1.7	265***
Night work	0.6	2.1	2.3	3.5	30***
Shift work	3.1	14.7	14.8	27.6	265***
Unsociable work days	8.3	15.9	19.2	19.0	83***
Low predictability	24.4	28.5	33.6	30.4	43***
Physical violence	0.7	3.6	1.7	0.9	45***
Bullying	22.7	22.1	22.5	23.7	1
Verbal abuse	25.7	31.0	26.2	13.3	94***
Demands for responsibility	35.1	38.1	29.2	32.3	62***
Biological exposure	12.1	30.6	34.5	25.7	228***
Chemical exposure	5.6	20.1	31.3	52.0	744***
Physical exposure	16.6	23.2	25.6	48.6	221***
Noise	10.8	17.7	20.0	37.0	276***
Thermic constraints	4.7	4.7	7.8	22.2	101***
Radiations	1.4	3.2	1.1	1.3	65***
Controlled air/space	28.7	21.4	16.0	14.4	135***
Biomechanical exposure	27.1	32.6	42.6	51.8	214***
Manual materials handling	5.8	22.6	32.8	47.4	605***
Postural/articular constraints	55.3	64.3	78.6	92.7	474***
Vibrations	0.4	0.6	1.3	8.0	319***
Driving	20.1	19.7	11.9	15.2	104***
Work injury					52***
0	98.8	97.3	95.6	93.9	
1	1.2	2.5	3.9	5.7	
2	0.0	0.2	0.4	0.4	
> =3	0.0	0.0	0.1	0.0	

Rao-Scott chi-square test

*: *p* < 0.05, **: *p* < 0.01, ***: *p* < 0.001

%: weighted %

Table 2 presents the association between occupation and work injury (after adjustment for age). Significant associations were found between occupation and work injury with strong occupational gradients. The RR of work injury associated with the occupation of blue-collar workers was 10 for men and 5 for women compared to professionals/managers. The stronger association between occupation and work injury among men than among women was confirmed by a significant interaction test among the whole sample (interaction test comparing blue-collar workers to professionals/managers between men and women significant at *p* = 0.024).

**Table 2:** Association between occupation and work injury

	Men (*N* = 26,432)	Women (*N* = 19,678)
RR (95% CI) (model 0)		
Associate professionals, technicians	4.0 *** (2.5; 6.5)	2.4 *** (1.5; 3.8)
Clerks, service workers	6.9 *** (4.6; 10.4)	3.9 *** (2.5; 6.1)
Blue-collar workers	10.5 *** (7.2; 15.4)	5.2 *** (3.2; 8.3)

RR adjusted for age (weighted Poisson regression analysis)

Professionals/managers: reference group

**: *p* < 0.05, **: *p* < 0.01, ***: *p* < 0.001

Table 3 presents the associations between the occupational exposures of chemical, biological, physical and biomechanical nature and work injury. When each exposure was studied separately (extended models 0), all exposures increased the risk of work injury except radiations and controlled air/space for both genders, vibrations for women and driving for men. When all occupational exposures were studied simultaneously (models 3 and 4), biological, physical and biomechanical exposures were significantly associated with work injury. Among the sub-dimensions of physical and biomechanical exposures, the associations of noise and manual materials handling with work injury were significant for both genders, and the associations of thermic constraints and vibrations were significant for men.

**Table 3:** Associations between occupational exposures and work injury: results from weighted Poisson regression analysis

	Men RR (95% CI)	Women RR (95% CI)
Extended models 0 (each factor separately)		
Biological exposure	**1.4** ** **(1.1; 1.8)**	**2.0** *** **(1.6; 2.4)**
Chemical exposure	**1.2** * **(1.0; 1.4)**	**1.7** *** **(1.4; 2.1)**
Physical exposure	**1.8** *** **(1.5; 2.3)**	**1.8** *** **(1.5; 2.3)**
Noise	**1.6** *** **(1.3; 2.0)**	**1.7** *** **(1.3; 2.1)**
Thermic constraints	**1.6** *** **(1.3; 1.9)**	**1.9** *** **(1.4; 2.5)**
Radiations	1.1 (0.7; 1.6)	1.2 (0.6; 2.3)
Controlled air/space	**0.8** * **(0.6; 0.9)**	0.9 (0.7; 1.2)
Biomechanical exposure	**1.4** *** **(1.2; 1.7)**	**1.6** *** **(1.3; 1.9)**
Manual materials handling	**1.7** *** **(1.4; 2.1)**	**2.3** *** **(1.8; 2.9)**
Postural/articular constraints	**1.7** *** **(1.3; 2.3)**	**1.5** ** **(1.1; 2.1)**
Vibrations	**1.6** *** **(1.3; 1.9)**	1.4 (0.8; 2.3)
Driving	1.2 (1.0; 1.4)	**1.3** * **(1.0; 1.7)**
Models 3		
Biological exposure	**1.3** * **(1.0; 1.7)**	**1.6** *** **(1.3; 2.1)**
Chemical exposure	1.0 (0.8; 1.2)	1.2 (0.9; 1.6)
Physical exposure	**1.7** *** **(1.4; 2.1)**	**1.6** *** **(1.2; 2.0)**
Biomechanical exposure	**1.3** ** **(1.1; 1.6)**	**1.4** ** **(1.1; 1.7)**
Models 4		
Biological exposure	**1.3** * **(1.0. 1.6)**	**1.5** ** **(1.2; 1.9)**
Chemical exposure	0.9 (0.7; 1.1)	1.1 (0.8; 1.5)
Noise	**1.3** ** **(1.1; 1.6)**	**1.4** ** **(1.1; 1.8)**
Thermic constraints	**1.3** *** **(1.1; 1.6)**	1.4 (1.0; 1.9)
Manual materials handling	**1.5** *** **(1.2; 1.7)**	**1.8** *** **(1.4; 2.3)**
Postural/articular constraints	1.3 (1.0; 1.7)	1.0 (0.7; 1.4)
Vibrations	**1.3** ** **(1.1; 1.5)**	0.9 (0.6; 1.6)

RR adjusted for age and occupation

Bold RR: significant at 5%

*: *p* < 0.05, **: *p* < 0.01, ***: *p* < 0.001

Model 3: Biological exposure + chemical exposure + physical exposure + biomechanical exposure

Model 4: Biological exposure + chemical exposure + noise + thermic constraints + manual material handlings + postural/articular constraints + vibrations

Tables 4 and 5 present the change in the RRs for each occupational group after inclusion of each factor/exposure (extended models 0). The following factors displayed significant contributions in the explanation of occupational differences in work injury: low reward, low esteem, low job promotion, workplace violence factors, and demands for responsibility among men, low decision latitude, low decision authority, low job promotion, shift work, unsociable work days, low predictability and physical violence among women, and low social support, low support from supervisors, job strain and isostrain for both genders. High psychological demands had significant but negative contributions and contributed to increase occupational differences in work injury. When psychosocial work factors with significant and positive contributions were considered simultaneously in models 1 and 2, significant contributions were found for models 1 among women (11–19%), and for models 2 for both genders (4–5% for men and 10–20% for women). These contributions were significant for clerks/service workers and technicians/associate professionals among both genders and for blue collar workers among women. In the models including each exposure separately (extended models 0), chemical, biological, physical and biomechanical exposures had significant contributions to the explanation of occupational differences in work injury. Noise, thermic constraints, manual materials handling and postural/articular constraints for both genders, and vibrations for men contributed to explain these differences. Controlled air/space had significant contributions among men, but this variable displayed significant protective associations with work injury (Table 3), and was not included in the final models. When the exposures with significant contributions were included simultaneously in models 3 and 4, their contribution was significant for all three occupational groups and for men (11–26%) and women (18–31%). When finally all factors and exposures with significant contributions were included in models 5 and 6, the contributions were significant for men (11–26%) and women (27–43%) and all three occupational groups.

**Table 4:** Contribution (%) of work factors to occupational inequalities in work injury: results for weighted Poisson regression analysis among men

Men	Associate professionals, technicians		Clerks, service workers		Blue-collar workers	
	RR	%	RR	%	RR	%
Extended models 0 (each factor separately)						
Low decision latitude	**4.0** ***	0.3	**6.8** ***	0.5	**10.4** ***	0.4
Low skill discretion	**4.0** ***	0.2	**6.9** ***	0.3	**10.4** ***	0.2
Low decision authority	**4.0** ***	1.2	**6.7** ***	1.9	**10.2** ***	1.5
High psychological demands	**4.2** ***	**−4.1** **	**7.5** ***	**−5.5** **	**11.7** ***	**−4.7** ***
Low social support	**3.9** ***	0.7	**6.5** ***	**1.1** *	**9.9** ***	**1.1** **
Low social support (from supervisors)	**4.0** ***	0.0	**6.6** ***	0.7	**10.2** ***	**0.8** **
Low social support (from colleagues)	**3.9** ***	0.1	**6.9** ***	0.1	**10.2** ***	0.1
Job strain	**4.0** ***	**1.3** *	**6.6** ***	**1.6** **	**10.2** ***	**1.0** **
Isostrain	**3.9** ***	0.8	**6.6** ***	**1.0** *	**10.0** ***	**0.6** *
Low reward	**3.9** ***	**2.4** **	**6.5** ***	**2.4** **	**10.2** ***	**1.1** **
Low esteem	**3.9** ***	**1.6** **	**6.6** ***	**2.1** **	**10.3** ***	**0.9** **
Job insecurity	**4.0** ***	0.0	**6.8** ***	−0.3	**10.5** ***	0.2
Low job promotion	**4.0** ***	**2.6** **	**6.8** ***	**2.4** **	**10.7** ***	**1.2** **
Long working hours	**4.1** ***	−1.8	**7.0** ***	−1.6	**10.7** ***	−1.4
Night work	**3.5** ***	−0.4	**7.1** ***	−0.9	**10.8** ***	−0.6
Shift work	**4.2** ***	−0.6	**7.2** ***	−1.1	**11.1** ***	−0.9
Unsociable work days	**4.0** ***	0.0	**6.8** ***	0.2	**10.5** ***	0.0
Low predictability	**4.0** ***	−0.1	**6.8** ***	0.5	**10.4** ***	0.0
Physical violence	**4.0** ***	0.3	**6.4** ***	**1.7** **	**10.4** ***	0.1
Bullying	**4.0** ***	**0.9** *	**6.7** ***	**1.2** **	**10.4** ***	0.4
Verbal abuse	**3.8** ***	**2.8** **	**6.1** ***	**5.4** ***	**10.5** ***	−0.3
Demands for responsibility	**4.0** ***	0.6	**6.9** ***	−0.5	**10.2** ***	**1.2** *
Model 1	**3.8** ***	1.8	**6.4** ***	1.9	**10.6** ***	−1.3
Model 2	**3.8** ***	**3.5** *	**6.0** ***	**4.7** *	**10.4** ***	0.8
Extended models 0 (each factor separately)						
Biological exposure	**3.9** ***	**1.9** **	**6.3** ***	**4.0** **	**10.1** ***	**1.2** **
Chemical exposure	**3.9** ***	2.4	**6.6** ***	**1.8** *	**9.5** ***	**4.2** *
Physical exposure	**3.5** ***	**8.9** ***	**5.9** ***	**7.3** ***	**7.5** ***	**14.5** ***
Noise	**3.7** ***	**5.7** **	**6.4** ***	**3.6** ***	**8.2** ***	**10.3** ***
Thermic constraints	**3.7** ***	**4.6** ***	**6.1** ***	**5.8** ***	**8.6** ***	**7.6** ***
Radiations	**4.0** ***	0.1	**6.9** ***	0.0	**10.5** ***	0.1
Controlled air/space	**3.9** ***	**2.0** *	**6.7** ***	**1.7** *	**10.0** ***	**2.3** *
Biomechanical exposure	**3.9** ***	**2.3** *	**6.5** ***	**3.1** **	**8.9** ***	**6.9** **
Manual materials handling	**3.5** ***	**8.3** ***	**5.8** ***	**8.0** ***	**7.4** ***	**14.1** ***
Postural/articular constraints	**3.8** ***	**5.1** **	**6.0** ***	**7.5** **	**8.7** ***	**9.2** **
Vibrations	**3.9** ***	**2.0** **	**6.7** ***	**1.2** **	**8.7** ***	**7.1** ***
Driving	**4.0** ***	1.0	**6.9** ***	0.0	**10.2** ***	1.3
Model 3	**3.4** ***	**10.7** ***	**5.4** ***	**11.9** ***	**6.8** ***	**18.5** ***
Model 4	**3.1** ***	**15.1** ***	**4.8** ***	**16.8** ***	**5.5** ***	**26.3** ***
Model 5	**3.3** ***	**10.9** ***	**5.2** ***	**12.4** ***	**7.3** ***	**14.9** ***
Model 6	**3.0** ***	**17.5** ***	**4.2** ***	**21.7** ***	**5.6** ***	**25.6** ***

Professionals/managers: reference group. RR indicates the RR of work injury for associate professionals/technicians (respectively clerks/service workers or blue-collar workers) in comparison with professionals/managers using various models

All models adjusted for age. Bold RR and contribution: significant at 5%. *: *p* < 0.05, **: *p* < 0.01, ***: *p* < 0.001

Model 1: Decision latitude + social support + reward + shift work + unsociable work days + predictability + physical violence + bullying + verbal abuse + demands for responsibility

Model 2: Decision authority + social support (from supervisors) + esteem + job promotion + shift work + unsociable work days + predictability + physical violence + bullying + verbal abuse + demands for responsibility

Model 3: Biological exposure + chemical exposure + physical exposure + biomechanical exposure

Model 4: Biological exposure + chemical exposure + noise + thermic constraints + manual material handlings + postural/articular constraints + vibrations

Model 5: model 1 + model 3

Model 6: model 2 + model 4

**Table 5:** Contribution (%) of work factors to occupational inequalities in work injury: results for weighted Poisson regression analysis among women

Women	Associate professionals, technicians		Clerks, service workers		Blue-collar workers	
	RR	%	RR	%	RR	%
Extended models 0 (each factor separately)						
Low decision latitude	**2.3** ***	5.5	**3.6** ***	**6.9** *	**4.6** ***	**7.5** *
Low skill discretion	**2.3** ***	3.2	**3.7** ***	4.3	**4.8** ***	4.5
Low decision authority	**2.2** ***	**6.9** *	**3.6** ***	**6.9** *	**4.7** ***	**6.8** *
High psychological demands	**2.6** ***	**−11.1** ***	**4.6** ***	**− 11.1** ***	**6.3** ***	**− 11.3** ***
Low social support	**2.3** ***	1.4	**3.9** ***	1.0	**5.0** ***	**2.6** *
Low social support (from supervisors)	**2.3** ***	1.7	**3.9** ***	0.4	**5.1** ***	**1.7** *
Low social support (from colleagues)	**2.3** ***	0.9	**3.9** ***	0.6	**5.1** ***	1.4
Job strain	**2.3** ***	**5.8** **	**3.6** ***	**5.5** ***	**4.8** ***	**5.2** **
Isostrain	**2.2** **	**3.8** *	**3.7** ***	**3.4** **	**4.9** ***	**4.2** **
Low reward	**2.2** ***	3.1	**3.8** ***	1.3	**5.1** ***	1.2
Low esteem	**2.3** ***	1.5	**4.0** ***	0.2	**5.2** ***	0.5
Job insecurity	**2.4** ***	0.7	**3.9** ***	−1.2	**4.8** ***	0.8
Low job promotion	**2.2** ***	3.0	**3.8** ***	**2.0** *	**5.1** ***	1.0
Long working hours	**2.6** ***	−5.6	**4.3** ***	−4.0	**5.7** ***	−3.3
Night work	**2.4** ***	0.1	**3.9** ***	0.1	**5.2** ***	0.1
Shift work	**2.2** **	**8.4** **	**3.6** ***	**5.3** **	**4.3** ***	**9.8** **
Unsociable work days	**2.3** ***	**3.6** *	**3.7** ***	**3.3** **	**4.9** ***	**3.0** **
Low predictability	**2.4** ***	1.9	**3.7** ***	**3.2** **	**5.0** ***	**2.2** *
Physical violence	**2.2** **	**3.7** *	**3.7** ***	**0.7** *	**5.2** ***	0.1
Bullying	**2.4** ***	−0.5	**3.9** ***	−0.1	**5.1** ***	0.4
Verbal abuse	**2.3** ***	**4.4** *	**3.8** ***	0.5	**5.6** ***	**−5.0** **
Demands for responsibility	**2.4** ***	0.8	**4.0** ***	−1.2	**5.2** ***	−0.3
Model 1	**1.8** *	**18.6** *	**3.1** ***	**11.3** *	**4.2** ***	9.3
Model 2	**1.8** *	**19.7** *	**3.1** ***	**12.0** **	**4.2** ***	**9.5** *
Extended models 0 (each factor separately)						
Biological exposure	**2.1** **	**14.5** **	**3.3** ***	**10.9** ***	**4.7** ***	**5.6** **
Chemical exposure	**2.2** **	**9.1** **	**3.4** ***	**10.1** ***	**4.0** ***	**15.2** ***
Physical exposure	**2.3** ***	**4.3** *	**3.7** ***	**3.7** **	**4.2** ***	**11.9** **
Noise	**2.3** ***	**4.1** *	**3.7** ***	**3.4** **	**4.4** ***	**8.5** **
Thermic constraints	**2.4** ***	−0.3	**3.9** ***	1.1	**4.5** ***	**6.9** **
Radiations	**2.4** ***	0.4	**3.9** ***	0.0	**5.2** ***	0.0
Controlled air/space	**2.4** ***	1.1	**3.9** ***	1.2	**5.1** ***	1.1
Biomechanical exposure	**2.3** ***	**2.7** *	**3.7** ***	**4.9** **	**4.6** ***	**6.8** **
Manual materials handling	**2.0** **	**16.6** **	**3.0** ***	**16.7** ***	**3.4** ***	**21.9** ***
Postural/articular constraints	**2.3** ***	4.3	**3.6** ***	**7.2** *	**4.5** ***	**9.8** *
Vibrations	**2.4** ***	0.0	**3.9** ***	0.2	**5.0** ***	1.5
Driving	**2.4** ***	0.0	**4.0** ***	−1.6	**5.3** ***	−0.7
Model 3	**1.9** **	**19.8** **	**3.0** ***	**18.3** ***	**3.4** ***	**23.5** ***
Model 4	**1.8** *	**25.5** **	**2.7** ***	**23.4** ***	**3.0** ***	**30.8** ***
Model 5	1.5	**39.1** *	**2.4** ***	**28.1** ***	**3.1** ***	**27.2** ***
Model 6	1.5	**42.9** *	**2.3** **	**30.3** ***	**2.9** ***	**30.7** ***

Professionals/managers: reference group. RR indicates the RR of work injury for associate professionals/technicians (respectively clerks/service workers or blue-collar workers) in comparison with professionals/managers using various models

All models adjusted for age. Bold RR and contribution: significant at 5%. *: *p* < 0.05, **: *p* < 0.01, ***: *p* < 0.001

Model 1: Decision latitude + social support + reward + shift work + unsociable work days + predictability + physical violence + bullying + verbal abuse + demands for responsibility

Model 2: Decision authority + social support (from supervisors) + esteem + job promotion + shift work + unsociable work days + predictability + physical violence + bullying + verbal abuse + demands for responsibility

Model 3: Biological exposure + chemical exposure + physical exposure + biomechanical exposure

Model 4: Biological exposure + chemical exposure + noise + thermic constraints + manual material handlings + postural/articular constraints + vibrations

Model 5: model 1 + model 3

Model 6: model 2 + model 4

The results from the KHB decomposition method showed that some factors played a significant role in the global contribution of the factors in models 1–6 (not showed): reward, esteem, physical violence, thermic constraints, and vibrations among men, predictability, and postural/articular constraints among women, and shift work, verbal abuse, biological exposure, physical exposure, noise, biomechanical exposure and manual materials handling for both genders, confirming the results from extended models 0.

## Discussion

### Main results

Strong occupational differences in work injury were observed for both genders, and still stronger for men than for women. Almost all psychosocial work factors and other occupational exposures displayed occupational gradients. The role of psychosocial work factors in explaining occupational differences in work injury was significant mainly for associate professionals/technicians and clerks/service workers and this role was stronger for women than for men. Differences between men and women were found for the contributing factors. The other occupational exposures of chemical, biological, physical and biomechanical nature contributed substantially to explain the occupational differences in work injury.

### Limitations and strengths of the study

The study used a large representative sample of the national French working population of employees, with weighted data and a good response rate, facilitating generalization of the findings. It can be noticed that the results were similar with or without including weights, although unweighted analyses led to more significant results; the presentation of the weighted results may thus be considered as a cautious approach. Men and women were analyzed separately, which is important in occupational epidemiology [38]. Indeed, in our study, there were differences in the prevalence of work injury and of exposure to work factors between genders. Furthermore, there were also differences in the magnitude of occupational inequalities in work injury and in the contributing factors between men and women. Occupational inequalities in work injury were studied, and the role of working conditions and occupational exposures was formally explored in the explanation of these inequalities, which is very rare in the literature. A large range of occupational factors and exposures was examined to provide a complete picture of working conditions. Well-established instruments were used to measure psychosocial work factors: the validated French versions of the JCQ (job strain model) and of the scale of reward (effort-reward imbalance model), facilitating comparisons with other studies. Other factors, understudied in the literature in this topic, such as job insecurity, workplace violence, working time and hours and demands for responsibility were also studied. The study also included other occupational exposures, that were measured by occupational physicians using their expert evaluation. We studied the contribution of each psychosocial work factor and each occupational exposure in the explanation of occupational differences in work injury. Models were also performed including all factors/exposures that displayed significant contributions. Indeed, there may be complex interrelations between factors/exposures; some factors may be causes or consequences of other factors. Because of these interrelations, it was not possible to sum the individual contributions provided by extended models 0. Thus, models 1–6 were useful to provide the global contribution of psychosocial work factors (models 1–2), of the other occupational exposures (models 3–4), and of all work factors together (models 5–6). Furthermore, additional analyses were done to disentangle the respective contribution of each factor in models 1–6, i.e. when all factors were taken into account. However, these additional results may be considered conservative given the complex interrelations between factors, especially regarding psychosocial work factors. Sensitivity analyses were also performed to adjust for two additional variables, working full/part time and years of work experience, that may play a role in the association between occupation and work injury. These analyses provided similar results confirming the robustness of the results. Finally, we used sophisticated statistical analyses to include weights, and to calculate confidence intervals and the significance of the contributions, that helped to select the factors in the final models.

A few limitations deserve to be mentioned. The study had a cross-sectional design, and the conclusions about statistical associations may not be causal. A reverse causality between work injury and occupation may be possible and may be explained by a selection effect called social selection. Indeed, employees with a work injury might have been selected in low-skilled occupations. However, social selection has been suggested to play a small role only in explaining social inequalities in other health outcomes [39]. A healthy worker effect may also be suspected in our study if low-skilled workers are more likely to have serious work injuries because of their working conditions that lead to death or exit from the labour market. This healthy worker effect may underestimate the association between occupation and work injury, as well as the contribution of occupational exposures/factors in the explanation of occupational differences in work injury. Work injury was self-reported and might not be as reliable as information from registers [40]. Psychosocial work factors were not all measured using validated questionnaires, and some factors may have been neglected. In addition, no information was available regarding duration of exposure, something that may lead to an underestimation of the contribution of occupational factors and exposures in social inequalities in health [41]. Finally, our study focused on occupational exposures in the explanation of occupational differences in work injury, and did not cover factors outside work.

### Comparison with the literature

The prevalence of work injury was found to be higher for men than for women in our study, in agreement with results in Europe reporting a prevalence for men that is twice the prevalence for women [2]. Strong occupational differences in work injury were found, and these differences were stronger for men than for women. In one of our previous studies using the SUMER 2003 survey data, we observed that the ORs for work injury associated with blue-collar workers compared to professionals/managers were 9.40 for men and 5.63 for women [7], which is consistent with the results found here using the SUMER 2010 data. Other studies underlined the strong social inequalities in work injury and showed that low-skilled or manual workers were more likely to have work injury [2–4]. Nevertheless, to our knowledge, there has been no previous study that evaluated the contribution of working conditions and occupational exposures in the explanation of these inequalities. One of our previous publications using the SUMER 2003 data explored the social inequalities in various health outcomes including work injury, but included a lower number of psychosocial work factors [7]. Its results showed that occupational exposures of chemical, biological, physical and biomechanical nature contributed to explain the occupational differences in work injury. The contributing physical and biomechanical exposures were noise, thermic constraints, manual material handling, postural/articular constraints, vibrations and driving. The total contributions of all these exposures, plus chemical and biological exposures, ranged from 6% to 33%, which is consistent with our present study based on the SUMER 2010 data. The contribution of psychosocial work factors (decision latitude, and especially decision authority, social support and workplace violence) accounted for 6–15% additional explained fractions. All these results are in agreement with the present results. The present study provided new findings regarding reward and its sub-dimensions, job promotion for both genders, and esteem for men, and working time and hours factors, shift work, unsociable hours and predictability, for women.

It may be worth noticing that the total contribution of working conditions and occupational exposures in explaining occupational differences in work injury was found to be 11–43% in our study. As our study had a good cover of working conditions (the SUMER survey is dedicated to the evaluation of all occupational exposures), it is unlikely that major aspects of working conditions may be missing and may account for a substantial unobserved explained fraction. Our study aimed at focusing on amendable factors at the workplace, thus it is possible that other factors may play a role in explaining occupational differences in work injury. For example, temporary employment, that is more prevalent among low-skilled workers, was found to be associated with work injury, and studies provided insight into potential mechanisms underlying this association such as length of experience and knowledge of workplace hazards [42]. Personal factors may also be contributing factors in social inequalities in work injury, such as for example chronic health problems [43], which also displayed strong social inequalities.

## Conclusion

Our study underlined the strong occupational differences in work injury and the still stronger differences among men. According to Eurostat, the most important risk factors of work injury are physical and biomechanical exposures at the workplace [2]. These exposures along with chemical and biological exposures may play a substantial role in explaining occupational differences in work injury. These types of exposures may contribute to these differences because they are risk factors for work injury and also because they display strong occupational gradients. Although most psychosocial work factors were found to be associated with work injury, their contribution in the explanation of occupational differences in this outcome may be lower, maybe because the occupational differences in these exposures were less marked. Comprehensive prevention policies oriented toward chemical, biological, physical, biomechanical and psychosocial work exposures may be useful to reduce the occurrence of work injury and the occupational differences in this outcome. Special attention should be given to low-skilled and manual workers. More studies are needed to confirm our results and also to identify the other factors that may contribute to explain the occupational inequalities in work injury.
